# A dynamic graph Hawkes process based on linear complexity self-attention for dynamic recommender systems

**DOI:** 10.7717/peerj-cs.1368

**Published:** 2023-05-09

**Authors:** Zhiwen Hou, Xiaojun Lv, Yuchen Zhou, Lingbin Bu, Qiming Ma, Yifan Wang, Fanliang Bu

**Affiliations:** 1 School of Information Network Security, People’s Public Security University of China, Beijing, China; 2 Institute of Computing Technology, China Academy of Railway Sciences Corporation Limited, Beijing, China

**Keywords:** Recommender systems, Dynamic graph, Hawkes process, Self-attention

## Abstract

The dynamic recommender system realizes the real-time recommendation for users by learning the dynamic interest characteristics, which is especially suitable for the scenarios of rapid transfer of user interests, such as e-commerce and social media. The dynamic recommendation model mainly depends on the user-item history interaction sequence with timestamp, which contains historical records that reflect changes in the true interests of users and the popularity of items. Previous methods usually model interaction sequences to learn the dynamic embedding of users and items. However, these methods can not directly capture the excitation effects of different historical information on the evolution process of both sides of the interaction, *i.e.*, the ability of events to influence the occurrence of another event. In this work, we propose a Dynamic Graph Hawkes Process based on Linear complexity Self-Attention (DGHP-LISA) for dynamic recommender systems, which is a new framework for modeling the dynamic relationship between users and items at the same time. Specifically, DGHP-LISA is built on dynamic graph and uses Hawkes process to capture the excitation effects between events. In addition, we propose a new self-attention with linear complexity to model the time correlation of different historical events and the dynamic correlation between different update mechanisms, which drives more accurate modeling of the evolution process of both sides of the interaction. Extensive experiments on three real-world datasets show that our model achieves consistent improvements over state-of-the-art baselines.

## Introduction

Dynamic recommendation is an important application of big data methods, which has been successfully applied in many fields, such as e-commerce, social media, health and medical services. In addition, dynamic recommendation can be implemented for public safety, such as predicting security events in a specific area (Liu et al., 2016b; Wu et al., 2016; Liu, Wu & Wang, 2017).

The real-world dynamic recommender system predicts the item that the user may interact with in the future by mining the time series information of the interaction between users and items. The two types of entities, users and items, can cover a variety of notions, *e.g.*, users could be customers in an ecommerce system, or accounts on social media; items could be products, posts, media produced or consumed by users. In real world scenarios, both user interest and item popularity may shift and evolve along with time. Besides, the historical interaction information (Hidasi et al., 2015; Li et al., 2017; Liu et al., 2016a; Wu et al., 2017) and the historical collaborative interaction information (Rendle, Freudenthaler & Schmidt-Thieme, 2010; Koren, 2009; Rendle et al., 2012) of users or items are proved powerful in making recommendation. The historical interaction information here refers to the historical interaction item (or user) sequence of the user(or item), and the historical collaborative interaction information refers to the second-order historical interaction node sequence obtained by using the high-order connectivity of the graph structure. Consequently, the key to building a dynamic recommender system is how to effectively combine the dynamic changes of users and items with their historical information.

At present, several approaches have been proposed to predict future items a user is likely to interact with, providing encouraging results (Hidasi et al., 2015; Wu et al., 2017; Covington, Adams & Sargin, 2016; Dai et al., 2016; Kumar, Zhang & Leskovec, 2019; Tan, Xu & Liu, 2016; Wang et al., 2020; Wu et al., 2019b; Xu et al., 2019). Often, however, the focus is on modeling users, while the historical interaction dynamics that provide a richer signal is overlooked (Wang et al., 2019). Therefore, recurrent neural networks (RNN) and other models suitable for sequences (Hidasi et al., 2015; Li et al., 2017; Liu et al., 2016a; Wu et al., 2017) are used to model the long-term dependencies of item sequences. Recently, some studies have shown that significant improvements to traditional methods can be achieved by building a user-item bipartite graph and modeling two types of entities at the same time (Dai et al., 2016; Kumar, Zhang & Leskovec, 2019). Unlike previous methods, JODIE (Kumar, Zhang & Leskovec, 2019) and other dynamic evolution models (You et al., 2019; Wang et al., 2016; Wu et al., 2019a) have employed mutually recursive RNNs that are more capable to model the user-item interaction dynamics. Dynamic graph collaborative filtering (DGCF) (Li et al., 2020) also uses the graph structure to model the collaborative information between nodes. However, these methods often ignore the influence of different historical interaction information and historical collaborative interaction information on the evolution process of the current interaction. Nonetheless, the Hawkes process can effectively capture this effect (Hawkes, 1971; Mei & Eisner, 2017), which assumes that historical events will increase the probability of future events to some extent, and this effect will decay with time. Therefore, the evolution process of user or item embedding can be effectively modeled by using this property. In this process, the users or items involved in the interaction when each link is formed can be considered to be determined by the influence of recent events. For example, in a product recommendation network, a particular product that has attracted a large number of buyers recently may attract more buyers in the near future.

Therefore, to effectively model the user or item embedding evolution process on the dynamic graph, we propose a Dynamic Graph Hawkes Process based on Linear complexity Self- Attention (DGHP-LISA) for dynamic recommender systems. First, we propose a new dynamic graph embedding update framework based on the Hawkes process, which can not only inherits the previous embeddings during the node embedding update, but also explicitly model the excitation effects of the evolution process of both sides of the current interaction through historical interaction information and historical collaborative interaction information. In addition, we design a new self-attention mechanism with linear complexity (LISA), which is efficient enough to be the backbone component of a deep network. Therefore, we add the LISA module in the time encoding of neighbor nodes and the feature fusion of node embeddings to capture the time correlation of different historical events and the dynamic correlation between different update mechanisms respectively. We evaluate the effectiveness of DGHP-LISA on three public datasets through experiments, and the results show that DGHP-LISA achieves the most advanced performance.

## Related Work

Different from static recommendation models such as matrix factorization (MF) and Bayesian personalized ranking (BPR), the main task of dynamic recommendation models is to capture the dynamic changes of users and items from historical and current interactions, so as to accurately predict the embedding trajectory of users or items over time to recommend items that users may interact with in the future. In the field of dynamic recommendation, traditional methods use a vanilla deep neural network (DNN) architecture to integrate manually designed or learned features into the recommendation model. For example, Covington, Adams & Sargin (2016) divided the recommender system of YouTube into two stages: recall and sorting, and the two-stage model used DNN with similar structure to provide personalized recommendations for a large number of users in millions of candidate videos.

As recurring patterns in user-item interactions are considered to be critical in recommending or predicting future activities, RNNs and its variants have been widely used in interaction prediction. Specifically, Hidasi et al. (2015) creatively applied RNN to session-based recommendation problems. The model predicts the next item clicked by the user according to the click sequence information of the user’s current session. As a variant of RNN, Wu et al. (2017) used long short-term memory (LSTM) to build a model to capture the dynamic changes of users and movies. Beutel et al. (2018) proposed the LatentCross model based on the gated recurrent unit (GRU), which incorporates multiple types of contextual information into the model by taking the dot product of the contextual features and the hidden states in the GRU.

Since activities that are close to an event in time are more likely to trigger such event than the ones that are far apart, encoding the time interval between activities is helpful for improving the performance of the recommender system. However, standard RNNs and its variants cannot handle the time intervals between historical events. Therefore, driven by the above requirements, many works have extended RNNs and their variants to fully account for time intervals (Zhang, 2019; Zhu et al., 2017). For example, Zhu et al. (2017) designed a time gate for LSTM, so that the model can not only effectively deal with serialized data, but also model time information very well.

However, all the above methods only simulate the changes of users’ interests through the user’s historical interaction sequence, while ignoring the evolution of the item. In order to solve this problem, many works have leveraged point process models and RNNs to jointly learn the dynamic embedding of users and items. Dai et al. (2016) applied the temporal point process to dynamic recommendation and design learnable intensity functions to model the dynamic impact between users and items. Kumar, Zhang & Leskovec (2019) defined embedding update operation and embedding projection operation to learn the dynamic embedding of users and items from a series of time interactions. Li et al. (2020) introduced dynamic graph into dynamic recommendation task for the first time, and extended JODIE by considering the second-order neighborhood information of dynamic interaction networks.

Our DGHP-LISA is inspired by the above work. However, these latest works do not take into account the impact of different historical interaction information and historical collaborative interaction information on the evolution of both sides of the interaction. Overall, the main contributions of the proposed model to dynamic recommendation scenarios are as follows:

1. We introduce Hawkes process and dynamic graph into dynamic recommendation scenarios at the same time to model the dynamic interaction and evolution between users and items;
2. We design a new self-attention with linear complexity and introduce it into the node embedding update framework based on dynamic graph Hawkes process. On the one hand, when time-encoding neighbor nodes, the LISA module can effectively model the time decay effects of different historical events on current events. On the other hand, when fusing features generated by different update mechanisms, the LISA module can effectively model the importance between update mechanisms by dynamically allocating weights;
3. Extensive experiments have been carried out on three public datasets. The experimental results show that, compared with the most advanced baseline, our DGHP-LISA achieves the best results.

## Preliminaries

### Dynamic graph recommendation

In this work, the goal of dynamic recommendation is to learn the representation of users and items from current interactions and history, and then predict the items that users are most likely to be interested in in the future. This “interest” is the relationship between the user and the recommended item. The graph is the basic data structure to express this relationship, which can well describe the relationship between users and items. Therefore, the graph structure is used to solve many problems in the field of dynamic recommendation (Li et al., 2020; Trivedi et al., 2019). Since a dynamic graph can naturally and effectively represent the interaction between users and items over time, we use a dynamic graph structure to model a dynamic recommendation problem.

Definition 1
: Dynamic graph. In essence, the dynamic graph here is a bipartite graph. Both the user and the item are nodes, and all interactions are located between the user and the item node. If the user interacts with the item at the time $t\in {\mathbb{R}}^{+},\forall t\in \left[ 0,T \right] $, it forms a dynamic graph ${G}_{t}= \left( U\bigcup V,{E}_{t} \right) $. Where *U* and *V* represent the set of users and items, respectively, and *U*⋃*V* is a finite set of all users and items. *E*_*t*_ is a finite set of interactions in *G*_*t*_, *i.e.,* a set of all interactions between users and items before time *t*. The dynamic graph ${{G}_{t}}_{0}= \left( U\bigcup V,{{E}_{t}}_{0} \right) $ of the initial time *t*_0_ consists of isolated nodes or snapshots of the dynamic graph, and the initial embeddings of users and items are initial feature vectors or random vectors.
Definition 2
: User-item interaction events. In dynamic recommendation, the user-item interaction event is a triple $ \left( u,v,t \right) $, which represents the formation of interaction $ \left( u,v \right) \in {E}_{t}$ between user node *u* ∈ *U* and item node *v* ∈ *V* at time *t*. Therefore, a dynamic graph can also be defined as a chronological sequence of user-item interaction events $I= \left\{ { \left( u,v,t \right) }_{n}:n=1,2,\ldots , \left\vert {E}_{T} \right\vert \right\} $.
Definition 3
: Static and dynamic embeddings. To encode long-term static properties and temporal dynamic characteristics of entities, all users and items are assigned static and dynamic embeddings. On the one hand, we denote the static embeddings of user *u* and item *v* at time *t* as $\bar {u}\in {\mathbb{R}}^{n}$ and $\bar {v}\in {\mathbb{R}}^{n}$, which do not vary with time. Following previous proposals (Zhu et al., 2017; Baytas et al., 2017), we use one-hot vectors as static embeddings for all users and items. On the other hand, we assign dynamic embedding represented by *u*(*t*) ∈ ℝ^*d*^ and *v*(*t*) ∈ ℝ^*d*^ to each user *u* and item *v* at the time *t*, which can model the behavior and attributes that change over time. While a new interaction joins the graph, user and item embeddings are updated by DGHP-LISA.

### Hawkes process

The Hawkes process is a linear self-excited point process, which assumes that historical events will have the excitation effects on current events. Its behavior is typically modeled by a conditional intensity function *λ*(*t*), the rate of event occurring at time *t* given the past events.

The conditional intensity function of Hawkes process can be expressed as follows:

(1) \begin{eqnarray*}\lambda (t)=\mu (t)+\eta \int \nolimits \nolimits _{-\infty }^{t}\kappa (t-s)dN(s)\end{eqnarray*}

where *μ*(*t*) is a constant greater than zero, which represents the base intensity at time *t* and does not change with the change of historical information. *κ*(⋅) is the kernel function of Hawkes process. Generally speaking, kernel functions have a variety of forms, including exponential kernel function, Gaussian kernel function and so on. Different kernel functions will affect the decay rate of the model to historical events, thus affecting the prediction ability of the model. For example, in the exponential kernel function, with the increase of time, the influence of historical events on current events gradually decreases. In the Gaussian kernel function, the decay rate becomes smoother with the increase of time. *N*(*t*) indicates the number of events that occurred before time *t*. *η* is a constant greater than zero, which is used to control the influence of the basic intensity and historical events. Since the Hawkes process is able to model the excitation effects between events to capture the influence of historical events on current events, it is well suited for modeling user and item representation in dynamic graph.

## Proposed Approach

**Figure 1:**
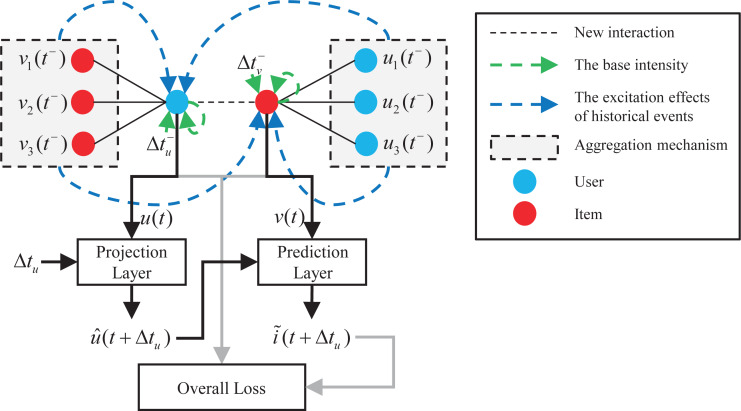
Overall architecture of the DGHP-LISA.

Figure 1 shows the overall architecture of the proposed model. The interactions between users and items form a dynamic graph over time. When a new user-item interaction event is observed, DGHP-LISA first learns the current embedding of the user and the item on the basis of the dynamic graph Hawkes process simultaneously. Secondly, inspired by the Kalman filter (Julier & Uhlmann, 1997; Hou & Bu, 2021), we capture the previously updated status and elapsed time through the projection and prediction layer to predict the future embedding of users and items. Thirdly, we calculate the *L*2 distance between the predicted item embedding and all other item embeddings, and then recommend items with the smallest distance to the predicted item embedding. Finally, we use the loss function to jointly optimize the dynamic embedding of users and items to make a more accurate prediction for the next item recommendation.

### Embedding update mechanism based on Hawkes process

#### Hawkes process on dynamic graph

The Hawkes process on the dynamic graph can model the dynamic embedding of users and items. Specifically, whether the user *u* or the item *v* participates in the formation of the interaction $ \left( u,v \right) $ at the time *t* can be quantified by the conditional intensity of this event.

(2) \begin{eqnarray*}{\lambda }_{u}(t)={\mu }_{u}(t)+\alpha \sum _{(u,{v}^{{^{\prime}}},{t}^{{^{\prime}}})\in {H}_{u}(t)}{\gamma }_{h{v}^{{^{\prime}}}}({t}^{{^{\prime}}})\kappa (t-{t}^{{^{\prime}}})+\beta \sum _{({u}^{{^{\prime}}},v,{t}^{{^{\prime}}})\in {C}_{u}(t)}{\gamma }_{c{u}^{{^{\prime}}}}({t}^{{^{\prime}}})\kappa (t-{t}^{{^{\prime}}})\end{eqnarray*}

(3) \begin{eqnarray*}{\lambda }_{v}(t)={\mu }_{v}(t)+\alpha \sum _{({u}^{{^{\prime}}},v,{t}^{{^{\prime}}})\in {H}_{v}(t)}{\gamma }_{h{u}^{{^{\prime}}}}({t}^{{^{\prime}}})\kappa (t-{t}^{{^{\prime}}})+\beta \sum _{(u,{v}^{{^{\prime}}},{t}^{{^{\prime}}})\in {C}_{v}(t)}{\gamma }_{c{v}^{{^{\prime}}}}({t}^{{^{\prime}}})\kappa (t-{t}^{{^{\prime}}})\end{eqnarray*}

where *μ*_*u*_(*t*) and *μ*_*v*_(*t*) are the basic intensity of the interaction events formed by nodes *u* or *v* at the time *t*, respectively, which are not influenced by historical events on *u* or *v*. The basic intensity determines the basic level of the current event, and it can also be understood as the expected probability of the event at the time *t*. The reasonable setting of the basic intensity can improve the prediction ability and explanation of the model. *α* and *β* are positive constants that control the influence of the basic intensity and historical events. If these two parameters are larger, then the impact of historical events on subsequent events will be more lasting. In practical application, we can balance the importance of historical events and the latest events by adjusting *α* and *β*.

*H*_*u*_(*t*) = {(*u*, *v*′, *t*′) ∈ *I*:*t*′ ≤ *t*} and *H*_*v*_(*t*) = {(*u*′, *v*, *t*′) ∈ *I*:*t*′ ≤ *t*} are the sets of historical interaction events of nodes *u* or *v* with respect to time *t*, respectively. Where *v*′ is the historical interaction neighbor of *u*, and *u*′ is the historical interaction neighbor of *v*. *γ*_*hv*′_(*t*′) and *γ*_*hu*′_(*t*′) respectively represent the influence degree of the historical interaction neighbor *v*′ or *u*′ on the current event at time *t*′.*C*_*u*_(*t*) = {(*u*′, *v*, *t*′) ∈ *I*:*t*′ < *t*} and *C*_*v*_(*t*) = {(*u*, *v*′, *t*′) ∈ *I*:*t*′ < *t*} are the sets of historical collaborative interaction events of node *u* or *v* with respect to time *t*, respectively. Where *u*′ is the historical cooperative interaction neighbor of *u*, *v*′ is the historical cooperative interaction neighbor of *v*. *γ*_*cu*′_(*t*′) and *γ*_*cv*′_(*t*′) respectively represent the influence degree of the historical cooperative interaction neighbor *u*′ or *v*′ on the current event at time *t*′. In the Hawkes process, the time decay effect of historical events on current events is modeled by kernel functions. The kernel function describes the decay process of the excitation effect of historical events with time. Therefore, the influence of historical events on the interaction probability of current events can be explained as the weighted sum of the excitation effect of historical events in time, in which the weight of time is determined by the kernel function. In the DGHP-LISA model, we use the kernel function *κ*(⋅) to model the time decay effect of historical events on current events, which can control the decay rate of historical events with time.

Next, combined with dynamic graph representation, the conditional intensity in Eqs. (2) and (3) is materialized. LISA(⋅) is a self-attention module with linear complexity, which can effectively model the importance between update mechanisms by dynamically allocating weights to different embedding update mechanisms. The conditional intensity can be generated from a transfer function *f* (Mei & Eisner, 2017; Panzarasa, Opsahl & Carley, 2009), *i.e.,*

(4) \begin{eqnarray*}{\lambda }_{u}(t)=f({LISA}\nolimits (u({t}^{-}),\Delta {t}_{u}^{-},{u}_{h}({t}^{-}),{u}_{c}({t}^{-})))\end{eqnarray*}

(5) \begin{eqnarray*}{\lambda }_{v}(t)=f({LISA}\nolimits (v({t}^{-}),\Delta {t}_{v}^{-},{v}_{h}({t}^{-}),{v}_{c}({t}^{-})))\end{eqnarray*}

where *u*(*t*^−^) and *v*(*t*^−^) are the latest embeddings of *u* or *v* before time *t*, respectively. $\Delta {t}_{u}^{-}$ and $\Delta {t}_{v}^{-}$ respectively represent the time interval between current time *t* and previous interaction time *t*^−^ of the node *u* or *v*. For dynamic graph, the node to be updated firstly inherits the influence of the previous state and elapsed time, which is used as the basic intensity to learn the embedding of users and items.

*u*_*h*_(*t*^−^) and *v*_*h*_(*t*^−^) represent the aggregate embedding of the historical interaction neighbors of *u* or *v* before the time *t*, respectively. The proposed model establishes a dynamic bipartite graph to simulate the interaction between user and item nodes, which means that the user’s historical interaction neighbors are the items that he or she has interacted with, and vice versa. In dynamic recommendation scenarios, the item that a user interacts with reflects the user’s recent interest to some extent. Correspondingly, users who are interested in a specific item can be regarded as a part of the item’s properties. Therefore, it is necessary to use the historical interaction neighbors of the user or item to learn its embedding.

*u*_*c*_(*t*^−^) and *v*_*c*_(*t*^−^) represent the aggregate embedding of the historical collaborative interaction neighbors of *u* or *v* before the time *t*, respectively. In the model, the historical collaborative interaction neighbors of the user or item node are the historical interaction neighbors of the other node involved in the interaction, which capture the collaborative relationship between users and items. Specifically, for a specific item, it may have been purchased by multiple users before the current interaction, and now a new user has purchased the item. It can be assumed that this new user has a collaborative relationship with the users who previously purchased the specific item. Therefore, the update of node embedding in dynamic graph takes into account not only the self-information and historical interaction of nodes, but also the structural information between nodes.

To sum up, the historical interaction aggregation embedding and historical collaborative interaction aggregation embedding of users or items before time *t* reflect the impact of historical events on current events, which is the key to simulate the excitation effect caused by historical events, combined with the basic intensity to learn the embedding of users and items.

#### Neighbor aggregation mechanism

It can be seen from Eqs. (4) and (5) that using the Hawkes process on the dynamic graph to model the dynamic embedding of users and items needs to consider the aggregate information of their historical interaction neighbors and historical collaborative interaction neighbors. To aggregate such information, let:

(6) \begin{eqnarray*}{u}_{h}({t}^{-})=\partial (v({t}^{-}),{{H}^{{^{\prime}}}}_{u}(t))\end{eqnarray*}

(7) \begin{eqnarray*}{u}_{c}({t}^{-})=\partial (u({t}^{-}),{{C}^{{^{\prime}}}}_{u}(t))\end{eqnarray*}

(8) \begin{eqnarray*}{v}_{h}({t}^{-})=\partial (u({t}^{-}),{{H}^{{^{\prime}}}}_{v}(t))\end{eqnarray*}

(9) \begin{eqnarray*}{v}_{c}({t}^{-})=\partial (v({t}^{-}),{{C}^{{^{\prime}}}}_{v}(t))\end{eqnarray*}

where ∂(⋅) is the aggregator function. *H*′_*u*_(*t*) and *H*′_*v*_(*t*) represent the historical interaction neighbor embedding set of nodes *u* or *v* with respect to time *t*, respectively. *C*′_*u*_(*t*) and *C*′_*v*_(*t*) represent the historical collaborative interaction neighbor embedding set of nodes *u* or *v* with respect to time *t*, respectively.

**Figure 2:**
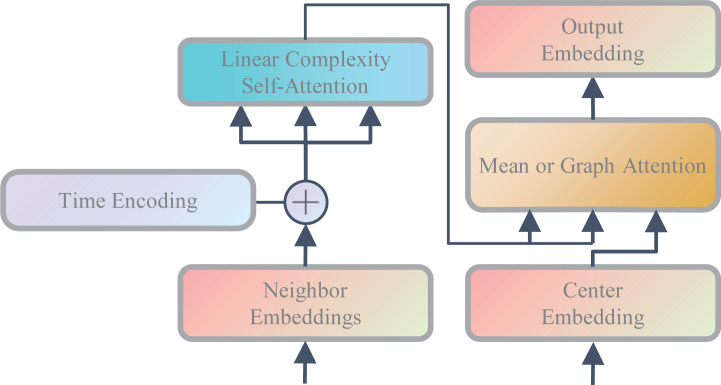
Neighbor aggregation mechanism.

(10) \begin{eqnarray*}{{H}^{{^{\prime}}}}_{u}(t)=\{ {{v}^{{^{\prime}}{^{\prime}}}}_{h}({t}^{{^{\prime}}}){|}{{v}^{{^{\prime}}{^{\prime}}}}_{h}({t}^{{^{\prime}}})={LISA}\nolimits ({v}^{{^{\prime}}}({t}^{{^{\prime}}})+h({t}^{{^{\prime}}})),(u,{v}^{{^{\prime}}},{t}^{{^{\prime}}})\in {H}_{u}(t)\} \end{eqnarray*}

(11) \begin{eqnarray*}{{H}^{{^{\prime}}}}_{v}(t)=\{ {{u}^{{^{\prime}}{^{\prime}}}}_{h}({t}^{{^{\prime}}}){|}{{u}^{{^{\prime}}{^{\prime}}}}_{h}({t}^{{^{\prime}}})={LISA}\nolimits ({u}^{{^{\prime}}}({t}^{{^{\prime}}})+h({t}^{{^{\prime}}})),({u}^{{^{\prime}}},v,{t}^{{^{\prime}}})\in {H}_{v}(t)\} \end{eqnarray*}

(12) \begin{eqnarray*}{{C}^{{^{\prime}}}}_{u}(t)=\{ {{u}^{{^{\prime}}{^{\prime}}}}_{c}({t}^{{^{\prime}}}){|}{{u}^{{^{\prime}}{^{\prime}}}}_{c}({t}^{{^{\prime}}})={LISA}\nolimits ({u}^{{^{\prime}}}({t}^{{^{\prime}}})+h({t}^{{^{\prime}}})),({u}^{{^{\prime}}},v,{t}^{{^{\prime}}})\in {C}_{u}(t)\} \end{eqnarray*}

(13) \begin{eqnarray*}{{C}^{{^{\prime}}}}_{v}(t)=\{ {{v}^{{^{\prime}}{^{\prime}}}}_{c}({t}^{{^{\prime}}}){|}{{v}^{{^{\prime}}{^{\prime}}}}_{c}({t}^{{^{\prime}}})={LISA}\nolimits ({v}^{{^{\prime}}}({t}^{{^{\prime}}})+h({t}^{{^{\prime}}})),(u,{v}^{{^{\prime}}},{t}^{{^{\prime}}})\in {C}_{v}(t)\} \end{eqnarray*}

where *h*(*t*′) represents the time embedding of time *t*′.*v*^′′^_*h*_(*t*′) and *u*^′′^_*h*_(*t*′) represent the historical interaction neighbor embedding of nodes u or v at time *t*′ after time encoding, respectively. *u*^′′^_*c*_(*t*′) and *v*^′′^_*c*_(*t*′) represent the historical collaborative interaction neighbor embedding of nodes u or v at time *t*′ after time encoding, respectively. As shown in Fig. 2, in order to enable the model to make full use of time information and capture the time decay effects of different historical events on current events, we add time embedding to all neighbor embedding and model the time correlation of different historical events through the LISA module.

For aggregator function ∂(⋅), we provide the following two candidate aggregator functions for use in neighbor aggregation:

1. Mean aggregator: It is a simple operator to calculate the average value of central node embedding and neighbor node embeddings, which can be regarded as a variant of GCN method (Hamilton, Ying & Leskovec, 2017; Kipf & Welling, 2016).
  (14) \begin{eqnarray*}{u}_{h}({t}^{-})= \frac{1}{1+ \left\vert {{H}^{{^{\prime}}}}_{u}(t) \right\vert } (v({t}^{-})+\sum _{{{v}^{{^{\prime}}{^{\prime}}}}_{h}({t}^{{^{\prime}}})\in {{H}^{{^{\prime}}}}_{u}(t)}{{v}^{{^{\prime}}{^{\prime}}}}_{h}({t}^{{^{\prime}}}))\end{eqnarray*}
  (15) \begin{eqnarray*}{u}_{c}({t}^{-})= \frac{1}{1+ \left\vert {{C}^{{^{\prime}}}}_{u}(t) \right\vert } (u({t}^{-})+\sum _{{{u}^{{^{\prime}}{^{\prime}}}}_{c}({t}^{{^{\prime}}})\in {{C}^{{^{\prime}}}}_{u}(t)}{{u}^{{^{\prime}}{^{\prime}}}}_{c}({t}^{{^{\prime}}}))\end{eqnarray*}
  (16) \begin{eqnarray*}{v}_{h}({t}^{-})= \frac{1}{1+ \left\vert {{H}^{{^{\prime}}}}_{v}(t) \right\vert } (u({t}^{-})+\sum _{{{u}^{{^{\prime}}{^{\prime}}}}_{h}({t}^{{^{\prime}}})\in {{H}^{{^{\prime}}}}_{v}(t)}{{u}^{{^{\prime}}{^{\prime}}}}_{h}({t}^{{^{\prime}}}))\end{eqnarray*}
  (17) \begin{eqnarray*}{v}_{c}({t}^{-})= \frac{1}{1+ \left\vert {{C}^{{^{\prime}}}}_{v}(t) \right\vert } (v({t}^{-})+\sum _{{{v}^{{^{\prime}}{^{\prime}}}}_{c}({t}^{{^{\prime}}})\in {{C}^{{^{\prime}}}}_{v}(t)}{{v}^{{^{\prime}}{^{\prime}}}}_{c}({t}^{{^{\prime}}}))\end{eqnarray*}
2. Attention aggregator: Inspired by the GAT (Veličković et al., 2017) model, it uses the attention mechanism to weighted summation of neighbor nodes.
  (18) \begin{eqnarray*}{u}_{h}({t}^{-})=\sum _{{{v}^{{^{\prime}}{^{\prime}}}}_{h}({t}^{{^{\prime}}})\in {{H}^{{^{\prime}}}}_{u}(t)}{\alpha }_{h}({t}^{{^{\prime}}}){{v}^{{^{\prime}}{^{\prime}}}}_{h}({t}^{{^{\prime}}})\end{eqnarray*}
  (19) \begin{eqnarray*}{\alpha }_{h}({t}^{{^{\prime}}})= \frac{\text{exp}(\text{LeakyRelu}({W}_{\alpha }[v({t}^{-}){|}{|}{{v}^{{^{\prime}}{^{\prime}}}}_{h}({t}^{{^{\prime}}})]))}{\sum _{{{v}^{{^{\prime}}{^{\prime}}}}_{h}({t}^{{^{\prime}}})\in {{H}^{{^{\prime}}}}_{u}(t)}\text{exp}(\text{LeakyRelu}({W}_{\alpha }[v({t}^{-}){|}{|}{{v}^{{^{\prime}}{^{\prime}}}}_{h}({t}^{{^{\prime}}})]))} \end{eqnarray*}
  (20) \begin{eqnarray*}{u}_{c}({t}^{-})=\sum _{{{u}^{{^{\prime}}{^{\prime}}}}_{c}({t}^{{^{\prime}}})\in {{C}^{{^{\prime}}}}_{u}(t)}{\alpha }_{c}({t}^{{^{\prime}}}){{u}^{{^{\prime}}{^{\prime}}}}_{c}({t}^{{^{\prime}}})\end{eqnarray*}
  (21) \begin{eqnarray*}{\alpha }_{c}({t}^{{^{\prime}}})= \frac{\text{exp}(\text{LeakyRelu}({W}_{\alpha }[u({t}^{-}){|}{|}{{u}^{{^{\prime}}{^{\prime}}}}_{c}({t}^{{^{\prime}}})]))}{\sum _{{{u}^{{^{\prime}}{^{\prime}}}}_{c}({t}^{{^{\prime}}})\in {{C}^{{^{\prime}}}}_{u}(t)}\text{exp}(\text{LeakyRelu}({W}_{\alpha }[u({t}^{-}){|}{|}{{u}^{{^{\prime}}{^{\prime}}}}_{c}({t}^{{^{\prime}}})]))} \end{eqnarray*}
  (22) \begin{eqnarray*}{v}_{h}({t}^{-})=\sum _{{{u}^{{^{\prime}}{^{\prime}}}}_{h}({t}^{{^{\prime}}})\in {{H}^{{^{\prime}}}}_{v}(t)}{{\alpha }^{{^{\prime}}}}_{h}({t}^{{^{\prime}}}){{u}^{{^{\prime}}{^{\prime}}}}_{h}({t}^{{^{\prime}}})\end{eqnarray*}
  (23) \begin{eqnarray*}{{\alpha }^{{^{\prime}}}}_{h}({t}^{{^{\prime}}})= \frac{\text{exp}(\text{LeakyRelu}({W}_{\alpha }[u({t}^{-}){|}{|}{{u}^{{^{\prime}}{^{\prime}}}}_{h}({t}^{{^{\prime}}})]))}{\sum _{{{u}^{{^{\prime}}{^{\prime}}}}_{h}({t}^{{^{\prime}}})\in {{H}^{{^{\prime}}}}_{v}(t)}\text{exp}(\text{LeakyRelu}({W}_{\alpha }[u({t}^{-}){|}{|}{{u}^{{^{\prime}}{^{\prime}}}}_{h}({t}^{{^{\prime}}})]))} \end{eqnarray*}
  (24) \begin{eqnarray*}{v}_{c}({t}^{-})=\sum _{{{v}^{{^{\prime}}{^{\prime}}}}_{c}({t}^{{^{\prime}}})\in {{C}^{{^{\prime}}}}_{v}(t)}{{\alpha }^{{^{\prime}}}}_{c}({t}^{{^{\prime}}}){{v}^{{^{\prime}}{^{\prime}}}}_{c}({t}^{{^{\prime}}})\end{eqnarray*}
  (25) \begin{eqnarray*}{{\alpha }^{{^{\prime}}}}_{c}({t}^{{^{\prime}}})= \frac{\text{exp}(\text{LeakyRelu}({W}_{\alpha }[v({t}^{-}){|}{|}{{v}^{{^{\prime}}{^{\prime}}}}_{c}({t}^{{^{\prime}}})]))}{\sum _{{{v}^{{^{\prime}}{^{\prime}}}}_{c}({t}^{{^{\prime}}})\in {{C}^{{^{\prime}}}}_{v}(t)}\text{exp}(\text{LeakyRelu}({W}_{\alpha }[v({t}^{-}){|}{|}{{v}^{{^{\prime}}{^{\prime}}}}_{c}({t}^{{^{\prime}}})]))} \end{eqnarray*}

where || is the concatenation operation and *W*_*α*_ ∈ ℝ^2*d*^ is a weight matrix.

In practical dynamic recommendation scenarios, due to the huge amount of data, neighbor aggregation needs to cost a high computational cost. Therefore, we select a fixed number of neighbors for aggregation and call the number of neighbor nodes selected as aggregator size.

#### Self-attention mechanism with linear complexity

Let *E*_*i*_ ∈ ℝ^*m*×*d*_*in*_^ and *E*_*o*_ ∈ ℝ^*m*×*d*_*out*_^ represent the input and output embedding of the LISA module respectively. *m* represents the number of embedding. *d*_*in*_ and *d*_*out*_ represent the dimensions of input and output embedding respectively. Let *Q* = *E*_*i*_*W*_*q*_, *K* = *E*_*i*_*W*_*k*_ and *V* = *E*_*i*_*W*_*v*_ denote the Query, Key and Value matrices generated by linear transformation on input embedding, respectively. Where *W*_*q*_ ∈ ℝ^*d*_*in*_×*d*_*k*_^, *W*_*k*_ ∈ ℝ^*d*_*in*_×*d*_*k*_^ and *W*_*v*_ ∈ ℝ^*d*_*in*_×*d*_*out*_^ are all weight matrices. The LISA module generates output embedding using the following self-attention operations based on linear complexity:

(26) \begin{eqnarray*}{E}_{o}=Q(\rho ({K}^{\top })V)\end{eqnarray*}

where *K*^⊤^ denotes the matrix transpose of *K*, and *ρ*(⋅) denotes the operation of applying softmax normalization for each row separately. The self-attention operation can be interpreted as first aggregating the features in the *V* matrix into *d*_*k*_ context vectors using the weights in the *ρ*(*K*^⊤^), and then reassigning the context vectors back to *m* using the weights in the *Q* matrix. The computational and memory complexities of this operation are *O*(*m*).

This self-attention operation is similar to the attention operation used by Chen et al. (2018) or Shen et al. (2021), but we do not use softmax standardization for *Q* matrix. Normalized *Q* matrix constrains the output embedding to a convex combination of context vectors, which may limit the expression ability of the self-attention mechanism. Therefore, we remove the softmax normalization of *Q* matrix, which allows the output embeddings to span the entire subspace of the *d*_*k*_ global context vectors.

#### Transfer function

The key to materialize the conditional intensity is to fit a transfer function *f* to the Hawkes process on the dynamic graph. In the past, softplus function and its variants are generally used as transfer function in the study of Hawkes process. To ensure that the output of the transfer function is positive, we instantiate *f* on the basis of Eqs. (7) and (8) as:

(27) \begin{eqnarray*}{\lambda }_{u}(t)=\RE \nolimits {LU}\nolimits ({SUM}\nolimits ({LISA}\nolimits (u({t}^{-}),\Delta {t}_{u}^{-},{u}_{h}({t}^{-}),{u}_{c}({t}^{-}))))\end{eqnarray*}

(28) \begin{eqnarray*}{\lambda }_{v}(t)=\RE \nolimits \text{LU}({SUM}\nolimits ({LISA}\nolimits (v({t}^{-}),\Delta {t}_{v}^{-},{v}_{h}({t}^{-}),{v}_{c}({t}^{-}))))\end{eqnarray*}

where the activation function uses ReLU. SUM(⋅) means that the features generated by different embedding update mechanisms are fused in a simple addition way.

#### The connection between transfer function and conditional intensity

A well chosen transfer function *f* , taking the dynamic graph representations of the node as input, is equivalent to the conditional intensity of the Hawkes process in Eqs. (2) and (3). In this section we formally show the connection.

First, we define the base intensity as a function of the self-information:

(29) \begin{eqnarray*}{\mu }_{u}(t)={f}_{u}(u({t}^{-}),\Delta {t}_{u}^{-})\end{eqnarray*}

(30) \begin{eqnarray*}{\mu }_{v}(t)={f}_{v}(v({t}^{-}),\Delta {t}_{v}^{-}).\end{eqnarray*}

Next, we define the influence of different historical events on current events as a function of historical interaction neighbors and historical collaborative interaction neighbors.

(31) \begin{eqnarray*}\sum _{(u,{v}^{{^{\prime}}},{t}^{{^{\prime}}})\in {H}_{u}(t)}{\gamma }_{h{v}^{{^{\prime}}}}({t}^{{^{\prime}}})\kappa (t-{t}^{{^{\prime}}})={f}_{\gamma }({u}_{h}({t}^{-}))\end{eqnarray*}

(32) \begin{eqnarray*}\sum _{({u}^{{^{\prime}}},v,{t}^{{^{\prime}}})\in {C}_{u}(t)}{\gamma }_{c{u}^{{^{\prime}}}}({t}^{{^{\prime}}})\kappa (t-{t}^{{^{\prime}}})={f}_{\gamma }({u}_{c}({t}^{-}))\end{eqnarray*}

(33) \begin{eqnarray*}\sum _{({u}^{{^{\prime}}},v,{t}^{{^{\prime}}})\in {H}_{v}(t)}{\gamma }_{h{u}^{{^{\prime}}}}({t}^{{^{\prime}}})\kappa (t-{t}^{{^{\prime}}})={f}_{\tau }({v}_{h}({t}^{-}))\end{eqnarray*}

(34) \begin{eqnarray*}\sum _{(u,{v}^{{^{\prime}}},{t}^{{^{\prime}}})\in {C}_{v}(t)}{\gamma }_{c{v}^{{^{\prime}}}}({t}^{{^{\prime}}})\kappa (t-{t}^{{^{\prime}}})={f}_{\tau }({v}_{c}({t}^{-})).\end{eqnarray*}

Given these building blocks, we rewrite the conditional intensity in Eqs. (2) and (3) as:

(35) \begin{eqnarray*}{\lambda }_{u}(t)={f}_{\lambda }(u({t}^{-}),\Delta {t}_{u}^{-},{u}_{h}({t}^{-}),{u}_{c}({t}^{-}))\end{eqnarray*}

(36) \begin{eqnarray*}{\lambda }_{v}(t)={f}_{\pi }(v({t}^{-}),\Delta {t}_{v}^{-},{v}_{h}({t}^{-}),{v}_{c}({t}^{-}))\end{eqnarray*}

where *f*_*λ*_ is a composite function of *f*_*u*_, *f*_*γ*_ and the summation, and *f*_*π*_ is a composite function of *f*_*v*_, *f*_*τ*_ and the summation. By choosing the right transfer function *f* , we further rewrite *f*_*λ*_ and *f*_*π*_ as the composition of *f* and the LISA module.

Finally, the conditional intensity can be expressed as follows:

(37) \begin{eqnarray*}{\lambda }_{u}(t)=(f\circ {LISA}\nolimits )(u({t}^{-}),\Delta {t}_{u}^{-},{u}_{h}({t}^{-}),{u}_{c}({t}^{-}))=f({LISA}\nolimits (u({t}^{-}),\Delta {t}_{u}^{-},{u}_{h}({t}^{-}),{u}_{c}({t}^{-})))\end{eqnarray*}

(38) \begin{eqnarray*}{\lambda }_{v}(t)=(f\circ {LISA}\nolimits )(v({t}^{-}),\Delta {t}_{v}^{-},{v}_{h}({t}^{-}),{v}_{c}({t}^{-}))=f({LISA}\nolimits (v({t}^{-}),\Delta {t}_{v}^{-},{v}_{h}({t}^{-}),{v}_{c}({t}^{-}))).\end{eqnarray*}

### Next item prediction

#### Project and predict the next item embedding

As shown in Fig. 1, in dynamic recommendation, user *u* interacts with item *v* at time *t*, and then interacts with item *i* at time *t* + Δ*t*_*u*_. Our task is to predict items that the user are most likely to interact with before time *t* + Δ*t*_*u*_, which is an analogy to link prediction problem in dynamic graph. Specifically, after updating the user and item embedding of time *t* by using the Hawkes process on the dynamic graph, the DGHP-LISA calculates the projected embedding $\hat {u}(t+\Delta {t}_{u})$ of user *u* and the predicted embedding $\tilde {i}(t+\Delta {t}_{u})$ of item *i* through the projection layer and prediction layer in turn. Then, we calculate the *L* 2 distance between the predicted item embedding and all other item embeddings, and then recommend items with the smallest distance to the predicted item embedding.

1. Projection layer: According to the method suggested in LatentCross (Beutel et al., 2018), based on the embedding *u*(*t*) of user *u* at time *t* and the elapsed time Δ*t*_*u*_, we incorporate time into the projected embedding *via* Hadamard product. The formula is as follows:
  (39) \begin{eqnarray*}\hat {u}(t+\Delta {t}_{u})=u(t)\odot (1+{W}_{pro}\Delta {t}_{u})\end{eqnarray*}
  where *W*_*pro*_ is used to convert Δ*t*_*u*_ into a time-context vector. We initialize *W*_*pro*_ by a 0-mean Gaussian. The vector 1 + *W*_*pro*_Δ*t*_*u*_ is used to scale the past user embedding. When Δ*t*_*u*_ = 0, the projected embedding is the same as the input embedding vector. The larger the value of Δ*t*_*u*_, the more the projected embedding differs from the input embedding and the projected embedding drifts over time.
2. Prediction layer: After obtaining the projected embedding $\hat {u}(t+\Delta {t}_{u})$ of user *u*, we use the prediction layer function to learn the future embedding of item *i* represented as $\tilde {i}(t+\Delta {t}_{u})$. We make this prediction based on the current user *u* and its interaction item *v* immediately before time *t* + Δ*t*_*u*_. The formula is as follows:
  (40) \begin{eqnarray*}\tilde {i}(t+\Delta {t}_{u})={W}_{pre}[\hat {u}(t+\Delta {t}_{u}){|}{|}\bar {u}{|}{|}v(t){|}{|}\bar {v}].\end{eqnarray*}
  As shown in Eq. (40), we use both the static and dynamic embeddings to predict the static and dynamic embedding of item *i* at time *t* + Δ*t*_*u*_, and *W*_*pre*_ represents the weight matrix.

#### Loss function

DGHP-LISA is trained to minimize the *L*2 distance between the predicted item embedding and the ground truth item’s embedding at every interaction. The loss function is as follows:

(41) \begin{eqnarray*}L=\sum _{(u,v,t)\in I}{ \left\| \tilde {v}(t)-[v({t}^{-})\parallel \bar {v}] \right\| }_{2}+{\lambda }_{u}{ \left\| u(t)-u({t}^{-}) \right\| }_{2}+{\lambda }_{v}{ \left\| v(t)-v({t}^{-}) \right\| }_{2}.\end{eqnarray*}

The first loss term minimizes the predicted embedding error. Since the items’ and users’ properties tend to be stable in a short time, the last two terms are added to regularize the loss and prevent the consecutive dynamic embeddings of a user and item to vary too much, respectively. *I* is a sequence of user-item interaction events in chronological order. *λ*_*u*_ and *λ*_*v*_ are scaling parameters, which are used to ensure the losses are in the same range.

### Optimization and training

In model training, we used a gradient-based Adam optimizer to optimize the parameters and use the same method of constructing batches as in Kumar, Zhang & Leskovec (2019) to speed up the training process. All interactions in each batch created by this method do not share any common nodes. DGHP-LISA works iteratively between the selection and removal steps. In the selection step, the method creates a new batch by selecting the largest set of edges at the earliest time. In the removal step, the previously selected edges are removed from the dynamic graph. Thus, each batch built by this method is parallelizable and maintains the sequential dependencies of all interactions.

**Figure 3:**
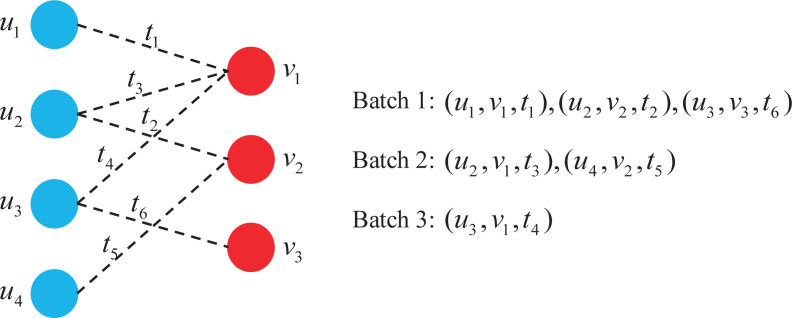
Batch construction in dynamic graph.

Figure 3 shows a dynamic graph of four users interacting with three items over time, where each dotted line represents the interaction associated with time.

As can be seen from Fig. 3, a total of three batches are constructed using this batch construction method, which is 50% less than when a single interaction is assigned to a batch. It is worth noting that each batch satisfies:

1. Each user and item appears at most once in every batch;
2. For the same user or item, earlier interactions are assigned to earlier batches.

## Experiments

### Dataset and preprocessing

In order to evaluate the performance of DGHP-LISA, we conduct experiments on three public datasets. The details of the datasets are shown in Table 1.

**Table 1:** The details of datasets.

**Data**	**Users**	**Items**	**Interactions**	**Action repetition**
Yelp	9,081	1,000	77,546	0.5%
LastFM	1,000	1,000	1293,103	8.6%
GTD	566	744	99,043	22.7%

1. Yelp: This dataset is a subset of Yelp’s businesses, reviews, and user data for use in personal, educational, and academic purposes. We first selected top 1,000 businesses with most number of reviews and users who made at least five reviews on the selected businesses. This resulted in 9,081 users and 77,546 interactions. In Yelp, a user interacts with the same business consecutively only in 0.5% interactions. In the experiment, this dataset is divided into training set, verification set and test set according to the proportion of 80%, 10% and 10%.
2. LastFM: This dataset is a widely used dataset that contains one month’s song listening information (Hidasi & Tikk, 2012). We selected all 1,000 users and the 1,000 most listened songs, resulting in a total of 1,293,103 interactions. Note that users only listened to the same song continuously in 8.6% of the interactions. The training set, validation set, and test set are divided in the same proportion as the Yelp dataset.
3. GTD: The global terrorism database is considered to be the most comprehensive database covering terrorist incidents in the field of global terrorism so far, including more than 200,000 terrorist incidents committed by about 3,000 terrorist organizations around the world since 1970. In order to provide early warning of terrorist attacks and reduce risks, we use DGHP-LISA to predict the next province or state where terrorist organizations may attack at a specific time. In the experiment, we deleted all events with uncertain dates, as well as events with unknown organization and location of the terrorist attacks. At the same time, we remove all the terrorist organizations with fewer than ten occurrences and all the locations where were attacked with less than ten occurrences. Finally, a total of 99,043 events were screened, of which a terrorist organization interacts with the same location of attack consecutively in 38% interactions. In the experiment, the data is split by time, and we train all models on the first 70% interactions, validate on the next 10%, and test on the last remaining interactions.

### Experimental setup

#### Evaluation metrics

We use the following two evaluation metrics for experiments:

1. Mean Reciprocal Rank (*MRR*) is the average of the reciprocal rank of the first positive example in all user recommendation lists. This indicator can measure the performance of the model with respect to the ranking list of items. Higher *MRR* score means the ground truth item tends to have higher rank positions in the predicted item lists. In some scenarios, such as ranking search results for specific users, the improvement of *MRR* can significantly improve the user experience, especially for items with high importance in ranking results. To calculate *MRR*, we use equation:
  (42) \begin{eqnarray*}MRR\text{=} \frac{1}{ \left\vert I \right\vert } \sum _{i\in I} \frac{1}{ran{k}_{i}} \end{eqnarray*}
  where *i* ∈ *I* represents traversing all interactions and *rank*_*i*_ represents the location of the ground truth item in the recommendation list for the *i*-th interaction.
2. *Recall@k* is the fraction of interactions in which the ground truth item is ranked in the top *k*. This metrics pays more attention to the integrity and diversity of recommendation results, and it is more suitable to be used in the recommendation system. Because the recommendation system needs to ensure that it covers as many items as possible in the user’s area of interest, so as to improve user satisfaction and participation. In some scenarios, such as e-commerce and news recommendation, the improvement of *Recall@k* can significantly improve the actual effect of the recommendation system, increase sales or user stickiness and so on. Formally, *Recall@k* is defined as:
  (43) \begin{eqnarray*}Recall@k\text{=} \frac{{n}_{hit}}{ \left\vert I \right\vert } \end{eqnarray*}
  where *n*_*hit*_ is the number of ground truth items that are among the top-*k* recommendation list, and $ \left\vert I \right\vert $ is the number of all test cases.

#### Parameter setting

We use Pytorch to implement DGHP-LISA, and the hyper-parameters are determined by the performance of the model on the verification set. For all algorithms, we use 128-dimensional dynamic embeddings and randomly initialize user and item embeddings with a Gaussian distribution with mean 0 and variance 1. Adam optimizer with learning rate 1e−3, *L*2 penalty 1e−5 is adopted in our model. Static embeddings all use one-hot vectors. The scaling parameters *λ*_*u*_ and *λ*_*v*_ in loss function are set to 1. All algorithms run for 50 rounds, and the corresponding test set is selected according to the best verification set. All experiments are run independently in the same experimental environment with Intel(R) Xeon(R) Gold 5118 host and NVIDIA Tesla V100-SXM2-32GB GPU. For comparison methods, we mostly use the default hyperparameters of the original paper.

### Baselines

To evaluate DGHP-LISA, we compare it with the following six baselines:

• LSTM (Hochreiter & Schmidhuber, 1997): It is a special RNN that captures long-term dependencies.
• Time-LSTM (Zhu et al., 2017): It adds two time gates to the standard LSTM to model the impact of time intervals on users’ current and long-term behavior.
• RRN (Wu et al., 2017): Based on the idea of matrix decomposition, it uses RNN to learn the dynamic embedding of users and items.
• DeepCoevolve (Dai et al., 2016): It is based on co-evolutionary point process algorithms. According to (Kumar, Zhang & Leskovec, 2019), 10 negative samples are used in each interaction.
• JODIE (Kumar, Zhang & Leskovec, 2019): It is a coupled RNN model that is used to learn the dynamic feature representation of users and items.
• DGCF (Li et al., 2020): It is a state-of-the-art model in dynamic recommendation problem, which is a new framework that utilizes dynamic graph to capture the collaborative and historical sequence relationships between users and items.

### Next item prediction experiment

For next item prediction, Table 2 compares the results of DGHP-LISA with six baselines on three datasets. In the experiment, DGHP-LISA uses a attention aggregator with an aggregator size of 20. The bold and underlined numbers mean the best and second-best results on each dataset and metric, respectively. “Improvement” denotes the performance improvement of DGHP-LISA over the best baseline.

From the experimental results, it can be observed:

1. DGHP-LISA outperforms all baselines on both metrics of the three datasets. Especially on the Yelp dataset, compared with the best baseline, the improvement of DGHP-LISA on *MRR* is 14.3% and the improvement on *Recall@10* is 23.6%. Also, compared with state-of-the-art baseline DGCF, the improvements on GTD, LastFM, and Yelp are in an increasing order, which are consistent with the repetitive action pattern in the datasets. The main reason for the improvement may be that DGHP-LISA explicitly captures the historical interaction information and historical collaborative interaction information. For example, users in Yelp datasets tend to comment on different businesses with similar categories, which results in a low action repetition. In this case, our model can consider both the historical interaction sequence of users and the similar interaction sequence of other users. The results show that DGHP-LISA can well deal with low action repetition situation.
2. DGHP-LISA performs significantly better than DeepCoevolve on three datasets. It shows that DGHP-LISA has a more appropriate conditional intensity function, which can better model the impact of historical events on the embedding update process of users or items.
3. DGHP-LISA and DGCF are superior to other baselines on three datasets, which indicates that the modeling of topological structure information is critical for learning dynamic graphs.
4. Considering the low density of user-item matrix in the three datasets, DGHP-LISA has certain advantages in dealing with the problem of data sparsity in the field of dynamic recommendation. First of all, the method makes use of the dynamic interactive information between the user and the item in the time series bipartite graph to make recommendations. This dynamic interactive information can provide more feature information and increase the accuracy of model prediction, so as to make up for the lack of data sparsity. Secondly, the DGHP-LISA models the changes of users’ interests and the evolution of item attributes at the same time by introducing Hawkes process, so as to better depict the co-evolution between users and items. It can be proved by experiments that this method can further enhance the representation of learning nodes in order to make full use of the limited data and make better recommendations.

### Experimental analysis and discussion

#### Ablation study

To verify the effectiveness of each module in the model, we implemented several variants of DGHP-LISA and conduct the next item prediction task on three datasets. In the experiment, DGHP-LISA uses a attention aggregator with an aggregator size of 20. First of all, we use only the basic intensity in the node embedding update framework based on the dynamic graph Hawkes process, and then gradually add the excitation effect of historical events, the time embedding of neighbor nodes, and the LISA module to form DGHP-LISA. The specific process is described as follows:

**Table 2:** Performance comparison of different methods on three datasets.

**Models**	**Yelp**		**LastFM**		**GTD**	
	***MRR***	***Recall@* 10**	***MRR***	***Recall@* 10**	***MRR***	***Recall@* 10**
LSTM	0.006	0.011	0.062	0.119	0.124	0.211
Time-LSTM	0.008	0.019	0.068	0.137	0.267	0.452
RRN	0.013	0.023	0.089	0.182	0.402	0.643
DeepCoevolve	0.004	0.009	0.019	0.039	0.051	0.085
JODIE	0.046	0.082	0.195	0.307	0.496	0.764
DGCF	0.077	0.144	0.321	0.456	0.509	0.785
DGHP-LISA	**0.088**	**0.178**	**0.328**	**0.474**	**0.510**	**0.803**
**Improvement**	14.3%	23.6%	2.2%	3.9%	0.2%	2.3%

**Notes.**

The bold and underlined values indicate the best and second-best results on each dataset and metric, respectively.

• variant *a* (basic intensity): We only use basic intensity to model in the node embedding update framework based on dynamic graph Hawkes process.
• variant *b* (+excitation effect of historical events): We add the modeling of the influence of different historical interaction events and historical collaborative interaction events on the current events.
• variant *c* (+time embedding of neighbor nodes): We add time embedding to all historical interaction neighbors and historical collaborative interaction neighbors.
• variant *d* (+ LISA): LISA module is added to the time encoding of neighbor nodes and the feature fusion of node embeddings.

Table 3 presents that the key modules in DGHP-LISA are effective for the next item prediction task. Specifically, modeling the influence of historical events on current events in the node embedding update framework based on dynamic graph Hawkes process is helpful to capture the excitation effects of different historical events. We add time embedding to all historical interaction neighbors and historical collaborative interaction neighbors, which is helpful to capture the time decay effect of different historical events on current events. We add the LISA module to the time encoding of neighbor nodes, which effectively captures the time correlation of different historical events. At the same time, we add the LISA module to the feature fusion of node embeddings , which effectively models the importance between update mechanisms. In conclusion, the ablation experiment presents the effectiveness of each module in our model.

#### Impact of different aggregator functions

In order to study the impact of different aggregator functions on the performance of the model, we test the effectiveness of mean and attention aggregator functions respectively. In the experiment, the size of the aggregator is 20. It can be seen from Table 4 that the attention aggregator is better than the mean aggregator on three datasets. It is proved that the attention aggregator can update the user or item embedding by selecting the information which is more beneficial to the prediction task in multiple historical interaction neighbors or historical collaborative interaction neighbors. However, the mean aggregator has the same weight when aggregating neighbor information, which may ignore the most influential nodes.

#### Impact of different aggregator sizes

In order to verify the impact of different aggregator sizes on the model performance, we use attention aggregator to set different aggregator sizes to evaluate the model. In the experiment, DGHP-LISA uses a attention aggregator, and the aggregator size is set to 20, 40, 60 and 80, respectively. As shown in Table 5, as the size of the aggregator increases, the performance of the model on three datasets decreases at first and then increases. When the size of aggregator is 20, the performance of the algorithm is the best. This shows that the increase of aggregator size may bring a lot of neighbor information redundancy, which is not helpful to modeling. Therefore, the accuracy of the model and the speed of training can be improved by reducing the size of the aggregator.

**Table 3:** Comparison of different variant models.

**Variants**	**Yelp**		**LastFM**		**GTD**	
	***MRR***	***Recall@* 10**	***MRR***	***Recall@* 10**	***MRR***	***Recall@* 10**
*a*	0.077	0.149	0.322	0.460	0.508	0.795
*b*	0.079	0.161	0.324	0.461	0.508	0.796
*c*	0.081	0.163	0.325	0.462	0.509	0.798
*d*	0.088	0.178	0.328	0.474	0.510	0.803

**Table 4:** Impact of different aggregator functions.

**Aggregator**	**Yelp**		**LastFM**		**GTD**	
	***MRR***	***Recall@* 10**	***MRR***	***Recall@* 10**	***MRR***	***Recall@* 10**
Mean	0.087	0.176	0.326	0.472	0.509	0.802
Attention	0.088	0.178	0.328	0.474	0.510	0.803

**Table 5:** Impact of different aggregator sizes.

**Aggregator size**	**Yelp**		**LastFM**		**GTD**	
	***MRR***	***Recall@* 10**	***MRR***	***Recall@* 10**	***MRR***	***Recall@* 10**
20	0.088	0.178	0.328	0.474	0.510	0.803
40	0.086	0.176	0.327	0.474	0.508	0.802
60	0.087	0.175	0.326	0.473	0.509	0.801
80	0.088	0.177	0.328	0.474	0.510	0.802

## Conclusions

We propose a new dynamic graph Hawkes process based on linear complexity self-attention and successfully apply it to the next item prediction problem. This model proposes an effective method to model the dynamic embedding of users and items in dynamic graph. Specifically, on the node embedding update framework based on Hawkes process, we add time embedding to all neighbor nodes to capture the time decay effects of different historical events on current events and model the excitation effects of different historical events. At the same time, we add LISA module to the time encoding of neighbor nodes and the feature fusion of node embeddings respectively, which effectively captures the time correlation of different historical events and the dynamic correlation between different update mechanisms. Finally, extensive experiments are carried out on three real-world datasets to prove that DGHP-LISA achieves the most advanced performance. In the future research work, a non-recursive network structure can be designed to further improve the training speed without affecting the accuracy of the model. Another direction of innovation is to explore the combination of other dynamic graph structure and recommender system except bipartite graph In addition, it is also possible to explore how to consider the impact of negative historical events on current events in DGHP-LISA to more accurately reflect user interest changes and item attribute evolution.

## Supplemental Information

10.7717/peerj-cs.1368/supp-1 Supplemental Information 1 Data and code

## Additional Information and Declarations

### Data Availability

The following information was supplied regarding data availability: The data and code are available in the Supplemental Files. Publicly available datasets were analyzed in this study. We evaluated our algorithm on three datasets: Yelp, LastFM, and GTD: - https://www.yelp.com/dataset; - http://ocelma.net/MusicRecommendationDataset/lastfm-1K.html; - https://www.start.umd.edu/gtd.
